# Factors influencing implementation of food and food-related waste audits in hospital foodservices

**DOI:** 10.3389/fnut.2022.1062619

**Published:** 2022-12-01

**Authors:** Nathan Cook, Jorja Collins, Denise Goodwin, Judi Porter

**Affiliations:** ^1^Department of Nutrition, Dietetics and Food, Monash University, Notting Hill, VIC, Australia; ^2^Eastern Health, Box Hill, VIC, Australia; ^3^BehaviourWorks Australia Health Programs, Monash University, Clayton, VIC, Australia; ^4^School of Exercise and Nutrition Sciences, Institute for Physical Activity and Nutrition, Deakin University, Geelong, VIC, Australia

**Keywords:** foodservice, hospital, food waste, audit, sustainability

## Abstract

**Background:**

Designing a food waste audit tool for novel hospital foodservice practice does not guarantee uptake. Intended users must be consulted to understand the tool’s feasibility and face validity. This study aimed to identify the perspectives of staff involved in the operation of hospital foodservices on (1) how an evidenced based consensus pathway food waste audit tool is perceived to translate into practice, and (2) to determine the factors that influence the completion of food and food-related waste audits within this setting.

**Materials and methods:**

Purposeful sampling was used to recruit staff with knowledge on the operation/governance of foodservices within hospitals in Victoria, Australia. Semi-structured interviews (*n* = 20) were conducted *via* Zoom to explore barriers and enablers to completing food and food-related waste audits and a previously published food waste audit tool. NVivo was used for inductive thematic analysis.

**Results:**

Three factors determined the completion of food and food-related waste audits in hospital foodservices, and each factor could be a barrier or an enabler; (1) capacity: the availability of time, labour and materials to complete an audit (2) change: staff resistance to audit procedures and how to gain their buy-in (3) processes, governance, and leadership: the opportunity for high level support, policy and structure to encourage waste audits if present. The consensus tool appeared to have face validity. Planning audit operations, conducting stakeholder meetings, providing education/training to foodservice team members, and facilitating communication between managers and staff were described to support consensus tool use and audit completion.

**Conclusion:**

The consensus tool can be used to support hospital foodservices to complete food and food-related waste audits, although it may need to be customised to be fit for purpose. Optimising the capacity, change management and processes, governance and leadership of the foodservice department may improve the experience and success of a food and food-related waste audit.

## Introduction

An estimated 40% of all food produced is lost or wasted globally (1). This has economic, environmental, and social consequences for society, including contributing nearly 10% of total carbon emissions, driving food insecurity and food scarcity, spawning community conflict, and costing the global economy around USD one-trillion annually (2). In response to the UN Sustainable Development Goals (SDGs) countries around the world have adopted their recommendation to halve global food waste by 2030 (SDG goal 12.3) (3). Measuring food waste through food waste audits and waste analytics is critical to achieving this goal as it allows industries to monitor their waste and confidently demonstrate progress over time (4). For example, the “*Target, Measure, Act*” campaign from the UK Waste and Resource Action Programme (WRAP) (5) asks food and drink businesses to set a food waste *target*, consistently *measure* their waste, and *act* to reduce this food waste. Measuring food waste also facilitates changes to practice to reduce the amount of waste generated and/or sent to landfill. The Australian National Food Waste Roadmap which lists 47 interventions predicts that measuring food waste is the intervention that has the second largest capacity to reduce food waste (2.69 million tonnes over 10 years) (6).

**FIGURE 1:**
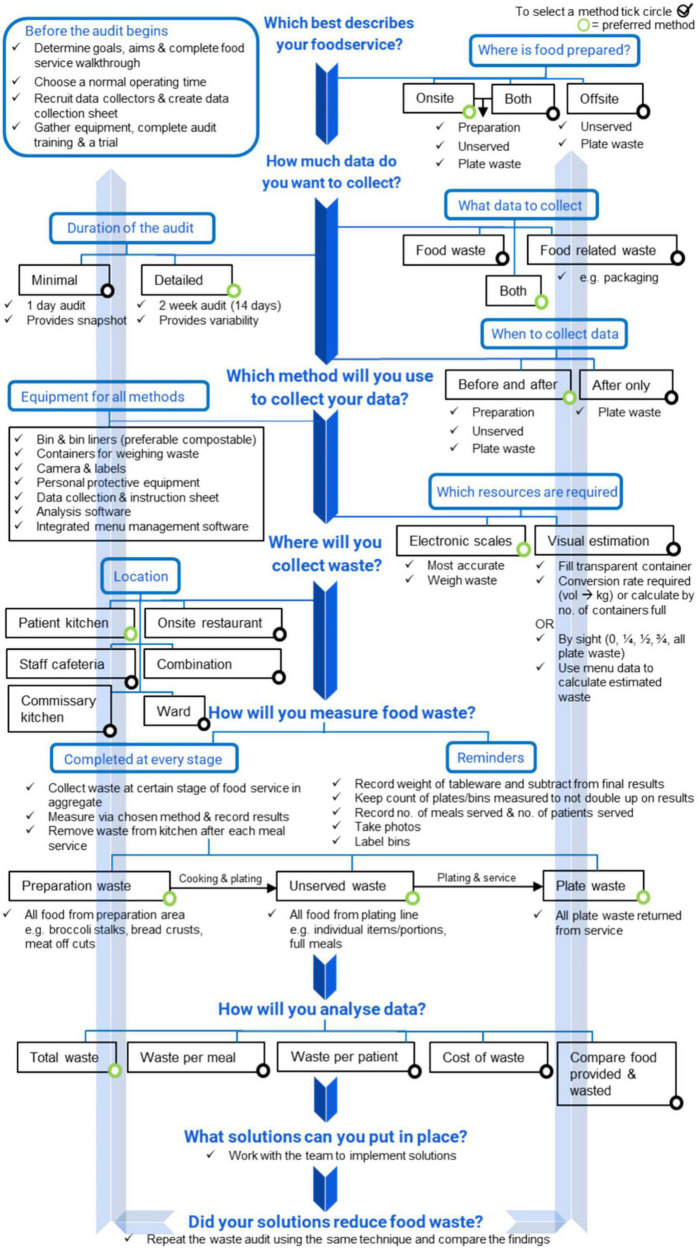
Hospital food waste audit consensus tool developed from the most common food waste audit methods identified in the systematic review of food waste audit methods in hospital foodservices originally presented in Cook et al. (10).

With these benefits in mind, measuring food waste could be transformative in the healthcare industry where there are high amounts of food waste; estimated as half of total hospital waste in some institutions (7). It arises due to a variety of reasons such as patients’ poor appetite, meal interruptions on the ward, food quality, portion sizes, and rigid ordering systems (8, 9). Aggregate food waste audits (which measure preparation waste, excess food, and plate waste) are important to quantify baseline waste, highlight problem areas or products within the foodservice, and monitor waste over time (10). A recent systematic review (10) consolidated 17 different food waste audit methods into a consensus pathway food waste audit tool that describes how to plan, conduct and analyse an audit in healthcare (Figure 1). The tool recommends that foodservices complete regular food waste audits for a duration of 2-weeks (14 days), collecting food and food-related waste (e.g., food packaging, plastic cutlery), before (preparation waste) and after (plate waste) meal times, including the waste from the plating line, and to measure waste using electronic scales.

However, there continues to be an evidence-practice gap when implementing evidence based practice change into healthcare settings (11, 12). Guidelines for complex settings such as hospitals are often developed and assumed to be adopted in practice without considering site readiness, local significance, or organisational goals (12). Previous research has reported that staff involved in hospital foodservice operations are aware of the food waste problem and want to implement strategies for measuring and reducing waste (13–15). However, limitations to measuring food and food-related waste exist and include minimal staff training, problems with data collection (e.g., faulty equipment and missing data) and audit method feasibility (10). Furthermore, kitchen staff completing a 1 day hospital food and food-related waste audit reported difficulty sorting their dish room waste because of staffing resources, safety, and space considerations (16). Other research suggests the challenges faced by foodservice staff are not unique to measuring food and food-related waste. For example, a study which explored the perspectives of hospital foodservice staff on their experiences of delivering a nutrition intervention, identified challenges with completing their existing work tasks due to strict time schedules, specific work role allocations, and the rigid foodservice structure (17).

The aims of this study were to identify the perspectives of staff involved in the operation of hospital foodservices on (1) how an evidenced based consensus pathway food waste audit tool is perceived to translate into practice, and (2) to determine the factors that influence the completion of food and food-related waste audits within this setting.

## Materials and methods

A qualitative description approach (18) was used where the authors approached the research from an interpretivist position (19). Interpretivism views knowledge as subjective and based on individual’s previous experiences. Realities are multiple and perceived to be socially constructed from the interactions between researchers and participants, to make meaning of the questions under investigation. Participants were hospital foodservice workers who were purposefully selected based on their wealth of knowledge and experience from their work role to describe and explain in depth the phenomenon under study (18). Inquiry through the use of semi-structured interviews allowed the opportunity for the researcher to interact with participants and obtain insightful information about what they perceived were barriers and enablers to completing food and food-related waste audits within their individual context. The study was approved by the Monash University Research Ethics Committee (Project ID: 28908) and was developed and reported following the Consolidated criteria for reporting qualitative research (COREQ) guidelines (20).

### Setting, participants, and recruitment

Participants sought to form the sample were staff with knowledge on the operation or governance of hospital foodservices. These key informants included foodservice workers and managers, foodservice dietitians, project coordinators, waste management staff, and sustainability officers. They were recruited from public hospitals in Victoria, Australia, using a maximum variation sample approach to include a varied selection of hospitals that may have different realities and experience or perceive the phenomena under study uniquely (21).

Using a random number generator (22) public hospitals in Victoria, Australia (23) were assigned a number 1–140, and clusters of 10 hospitals were invited fortnightly to participate in the study. To recruit the desired key informants for interviews, hospital administrators were contacted by phone to ask for operations managers email addresses. Operations managers were then contacted to identify and request contact details of possible participants, which were shared (with consent) to the research team. A consent form, organisational permission letter and explanatory statement were provided to contacted participants. When these were returned a one on one interview was scheduled. At the end of interviews a snowballing sampling strategy was utilised, whereby participants were asked to identify (if possible), another participant with knowledge on the research topic, and were requested to ask this person to contact the research team if they were interested in participating (24).

Recruitment, data collection and analysis were completed concurrently. During data analysis a combination of information power (considering this study’s broad aim, sparse specificity, use of theory, lower dialogue quality, and cross-case analysis strategy) (25) and evidentiary adequacy (ensuring an adequate amount, variety, interpretative, disconfirming, and discrepant evidence is collected) (26) were utilised to determine when a sufficient sample size of hospitals had been reached. The appropriate sample size was deemed satisfactory by the research team through the assistance of the maximum variation sampling technique (21) as it facilitated sampling a wide range of multiple realities (different hospitals) and diverse experiences (different participant roles) helping to reach information power (25). Therefore, a larger sample of hospitals was sought compared to other qualitative studies (9, 13, 27, 28) in this area of research as there is a finite number of potential participants within each hospital (as hospitals usually only contain one foodservice dietitian and foodservice manager). Recruitment of hospitals and therefore identification of important key informants was difficult due to the interruptions of COVID-19 with low response rates from invited participants.

### Data collection

The semi-structured interview schedule (Table 1) developed by the researchers for this study consisted of open-ended questions to explore barriers and enablers toward completing food and food-related waste audits. Prior to data collection, pilot interviews were completed with nine individuals (four dietitians, four foodservice dietitians, and one food safety and quality coordinator) to test the interview protocol, gauge participant understanding and refine the protocol if required. The major alteration for the interview protocol was providing participants the consensus pathway food waste audit tool (Figure 1) (10) and an explanation of how it was designed for use, in advance of their interview.

**TABLE 1:** Semi-structured interview protocol to explore staff involved in the operation of hospital foodservices perspectives on how an evidenced based consensus pathway food waste audit tool is perceived to translate into practice, and to determine the factors that influence the completion of food and food-related waste audits within this setting.

Main semi-structured interview questions
*1. Can you please tell me about your role, how long you have been in this role and how it is involved in the operation of the hospital foodservice?* *2. What do you see as current and anticipated barriers toward regular food waste audits in hospital foodservices?* *3. What can you see as current and anticipated enablers toward regular food waste audits?* *4. Reflecting on our discussion of barriers and enablers to hospital foodservice food waste audits, what strategies do you think could be used to best roll out a food waste audit in your hospital?* *5. What do you think of the decision tree pathway (referring to pathway included within previous publication, which was shared with participants) (10)? Would you change anything?* *6. How, or in what way, could the decision tree pathway support food waste audits in your foodservice?* *7. When you think back to what we have discussed today is there anything you would like to add before we conclude the interview?*
**Example prompt questions**
*- Why do you think these barriers are in place?* *- How do they effect the completion of food waste audits?*

All interviews were conducted and audio recorded *via* zoom (Version 5.5, Zoom video communications, California) during the period August to November 2021 by one researcher, a Ph.D. candidate and Accredited Practising Dietitian who has previously worked in foodservices and had prior research experience on the topic of food waste audits. Before interviews began, this researcher explained their position and relationship to the project, and confirmed the participant’s understanding of the interview topic, data confidentiality and consent. Demographic information (e.g., name, age, gender, job title, and time in position) were collected for descriptive purposes and to verify data sources during analysis. This research was completed during Victoria’s 6th COVID-19 lockdown period, thus to reduce participant burden interviews were not repeated and transcripts were not member checked. However, clarification of participant responses or contribution of additional information by participants occurred by email exchange. Additionally, to ensure the researcher provided their full attention to the participant field notes were not taken. Therefore to demonstrate researcher reflexivity during data collection, the interviewer and two other research team members met fortnightly to discuss the concurrent findings (peer debriefing) (29).

### Data analysis

Demographic information were analysed and reported using descriptive statistics in Microsoft Excel (Version 16.0). Audio recorded interviews were auto-transcribed using Otter.ai (Version 2.1.52, California),^1^ and transcripts were checked for accuracy by the primary researcher by concurrently listening to recorded audio and manually editing the transcripts. Inductive thematic analysis using a semantic approach proposed by Braun and Clarke (30) was completed by the primary researcher using NVivo (NVivo, QSR International, Victoria). One researcher (NC) conducted initial coding and assigned codes as either “barriers” or “enablers.” Next codes with similar meanings or ideas were categorised into broader factors underneath the “barriers” or “enablers” headings. This thematic analysis technique was guided by the approach of previous qualitative studies exploring barriers and enablers to different guidelines and interventions (31–34).

## Results

Twelve hospitals participated out of the 70 contacted (17% response rate) where a majority of hospitals were unable to be recruited due to unavailable operations managers email addresses, lack of response, and eligible participants declining the invitation due to time commitments. From the 12 hospitals, 21 participants were interviewed with five of these recruited through snowball sampling. One of the 21 participants withdrew their data post-interview, leaving 20 participants from 11 hospitals for data analysis.

Of the participants, the average (±standard deviation) age was 44 ± 11 years, with a majority (*n* = 6, 30%) between the ages of 31–40 years. Sixty percent of the participants were female. Participants had worked in their current role between 2-months and 25 years, mostly (*n* = 15, 75%) for less than 5 years. The most prominent role reported was foodservice dietitian (*n* = 4), followed by hotel services coordinator. The other 14 positions reported had varying responsibilities, including: cooking food, stock management, allergy control, recipe development, management of sustainability projects, and training of staff. The hospital size represented ranged between 18 and 600 beds, with most hospitals ranging from 100 to 300 beds. The most common foodservice production method was cook chill (*n* = 5), consistent with the practices of Victorian public hospitals, followed by cook fresh (*n* = 2), cook freeze (*n* = 1) and the remaining kitchens using combinations of cook chill, cook fresh and cook freeze (*n* = 3) (35). Five health services reported completing a food waste audit previously, measuring either unserved or plate waste. Interview length ranged from 50 to 94 min.

The following results discuss participants’ perspectives of the consensus pathway food waste audit tool including their general reflections and preliminary thoughts, suggested recommendations for change in design and how they perceive the tool would be used in practice.

### Strategies to implement the consensus tool

Preparing for an audit and introducing the concepts to staff were described by participants when asked how they would support the execution of a food waste audit within their hospital. Participants suggested that understanding the goals of the audit, assessing their current practice before auditing, planning audit logistics, conducting meetings, providing education to the foodservice team, and facilitating communication between managers and staff about the audit process were all important to ensure a food waste audit was completed.

*“it’s just figuring out what we actually want to get out of it, for starters, figuring out what we want to do, what goals, and what we want to get out of the audit.”* (Participant 8, Hotel services coordinator)

Two participants (Participant 2, Foodservice project officer and Participant 4, Food safety supervisor) suggested that creating a data entry sheet to record waste volumes would be helpful in addition to the consensus tool, as it would avoid them needing to create this themselves. Moreover, one participant (Participant 11, Group management support services) suggested that introducing an audit at the same time as another foodservice system change (such as a new menu or the integration of a new electronic menu system which supported data entry) would enhance the implementation of a food waste audit compared to having multiple different system changes occurring over time. This point was not raised by others.

### Perceptions of the consensus pathway food waste audit tool

The majority of the participants were supportive of the tool and believed it was detailed, supported understanding of concepts, encouraged different thinking and would facilitate decision making for the completion of a food waste audit.

*“I mean, I love your food waste audit tool. And it gets to the real nuts and bolts of where we’re wasting and allows us to then look at it and then just see where we’re wasting food and perhaps then drill down as to why.”* (Participant 12, Foodservice dietitian)

Some participants perceived the tool as busy and confusing, while others commented that its use would be individual and specific, with a higher level of knowledge needed to use it in practice. Recommendations to change the structure and content of the tool were provided by 14 participants. Several respondents advised that they would alter the tool to suit their individual needs; for example, one participant focused their recommendations around providing more explanation on how to complete the audit, and to include detailed information why certain areas of food waste needed to be considered.

*“I just need more info [Information] because all this info is good to know, this is what I need to do, but it doesn’t show me how to do it, you get what I mean?. so probably just mention a little bit more like preparation waste is for this, and why are you looking at that? And why are you looking at unserved waste? And why are you looking at plate wastage? I guess, a little bit more detail. I think you have to think about who is the audience…”* (Participant 10, Foodservice dietitian)

When asked how the tool would support participants to complete a food waste audit, the most common response was that the tool supported appropriate decision-making processes to be able to complete a food waste audit successfully. Several participants reported that the tool was ready to use and explained how they would use it in practice.

*“I think it’s great, because it gives a tool for people that don’t have that prior knowledge to use, so I can give this to the foodservice supervisor, and say, I think if you’re doing an audit, this is what you need to consider before you just jump in and do an audit…. And they can use this to actually ask themselves what do I need to do? What do I need to consider?”* (Participant 17, Foodservice dietitian)

Other comments were focussed toward using the tool for education purposes, with one participant (Participant 12, Foodservice dietitian) suggesting the tool could act as a starting point and training guide to prepare students for a food waste audit project.

*“I think it’s just a really neat way of preparing for a food waste (audit). So I could imagine showing this to a student and saying, what are we going to measure? Are we going to measure everything? Or are we just going to measure plate waste? Are we just going to measure preparation waste? and then following it down I think would work really well. So it could be very useful as a training tool.”*

**FIGURE 2:**
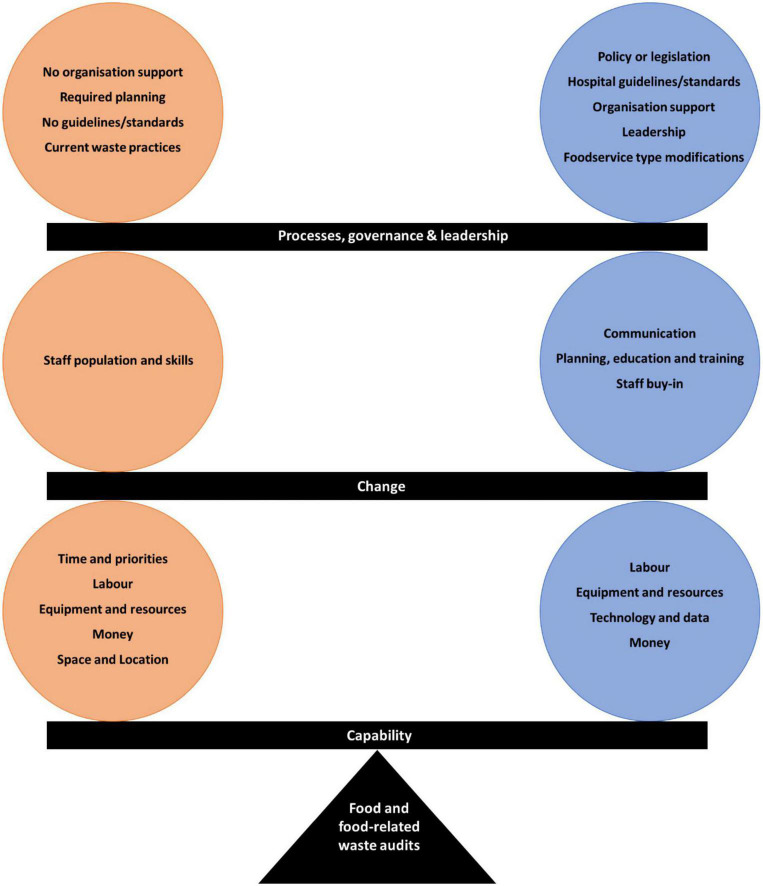
Participant reported barriers and enablers to the completion of food and food-related waste audits. Orange circles represent barriers and blue circles represent enablers. White text are the major factors and the black text are individually generated codes.

The subsequent results describe the findings for the second aim of this study. There were three factors which determined the completion of food and food-related waste audits in hospital foodservices; (1) capacity: the availability of time, labour and materials required to complete an audit (2) change: staff resistance to audit procedures and how to gain their buy-in (3) processes, governance and leadership: the opportunity for high level support, policy and structure to encourage waste audits if present. The barriers and enablers for each of these factors are presented below and are connected whereby one suggested barrier can be solved by a recommended enabler and vice versa (Figure 2).

### 1. Capacity

Time was identified as the largest barrier to conducting food waste audits in hospitals and was mentioned by all participants, in part attributable to the study being conducted during the COVID-19 Delta outbreak. Several participants explained how foodservices run on a strict time schedule and the inclusion of additional tasks required time to be allocated within rostered hours. Participants perceived that there was no time left in their scheduled work day to consider completing a food waste audit due to the large nature and detail of the task, day to day interruptions, and time constraints of usual practice. Moreover, participants reported that it is hard to justify a food waste audit when other tasks such as allergen management and meeting government food standards take priority. Participants also commented on the expected regularity (more than once a year) of audits (10) and that repeating the audit regularly would also not be feasible. One participant (Participant 13, Sustainable food systems dietitian) who had previously completed an audit, described that it took 6-months to plan the audit, complete it, and analyse the data.

Labour was also recognised as a one of the largest barriers to completing food waste audits. A food waste audit was labelled as labour intensive, needing a lot of staff resources to collect, sort and measure waste. Foodservices were perceived as *“an understaffed department”* that experiences considerable unplanned leave, with staffing numbers sometimes barely meeting requirements to run day to day operations. For example, one participant (Participant 1, Support service manager) advised that her department has minimal full time staff and are reliant on part-time or casual staff. In addition, time pressures were frequently reported by participants. This resulted in the question of *“who are we going to get to do it?”* being stated often by participants. They described that they would need to hire extra staff to complete audit tasks, find extra labour hours (time) or re-allocate their current workforce to meet audit requirements. The associated funding needed to pay staff was also a related issue.

*“And the labour, so if I was going to do this at lunchtime, I’d actually need another two people assisting, like checking as the trolleys come in because there’s time limits on the strip down of the trolleys to get everything ready for the next meal period. So, there would be me, let’s say the supervisor and then we’d need another body to assist. And so that would be the same if we’re going to do it for breakfast, lunch and dinner.”* (Participant 20, Facilities services manager)

Participants felt staff needed a deep knowledge and understanding of the process to execute the audit correctly. One participant (Participant 6, Foodservice manager) suggested her staff did not have the necessary skills or knowledge to complete the audit but others felt their staff were capable.

The belief of participants was that the lack of foodservice specific equipment and materials were also a hindrance to food waste audit completion. These included practical tools for data collection such as technology (spreadsheets, artificial intelligence, electronic foodservice systems), forms and templates, as well as training materials to support this process. Other items required for completing industrial-scale food waste audits, such as weighing scales and bins, were not readily available within the organisations who were represented. These items were important because those interviewed, as well as their staff, did not have the knowledge to design their own or procure these resources.

*“What, I was trying to say is, that is it something we can just adopt, and use? Rather than trying to create something new again, for example, you mentioned here in the decision tree tool the resources required are electronic scales. But it did not mention what kind of electronic scales? What are you referring to? How big or small? Are you talking about mini ones where we just weigh? Or are you talking about the big one? So from my experience, I know that we are talking about the huge one. In terms of, where can we get it from? So be as detailed as possible…”* (Participant 10, Foodservice dietitian)

Additionally, some participants from rural sites suggested that the location of the hospital and the position of the kitchen reduced the practicality of completing an audit due to the design of the kitchen providing little space to collect waste.

An increase in labour was the most prominent enabler described by interview participants. To aid audit completion, participants suggested multiple solutions including creating a new role solely focussed on completing food waste audits *via* increasing full time equivalent hours, using casual employees, or splitting tasks up between current staff. However, this would require alterations to current staff scheduling practices. Participants also placed value in having tertiary level healthcare students complete food waste audits. Other suggestions included collaborating with other departments or hiring an external person to undertake the audits.

*“or maybe getting someone who’s qualified to do it, a bit like an external food safety auditor…. maybe the same thing, maybe you have a food waste auditor, that comes out periodically and does the audits for you and presents the results and says these are the issues, and this is what you have to address.”* (Participant 4, Foodservice dietitian)

Resources were also perceived as enablers toward completing food waste audits. Equipment to facilitate the audit process, including scales, bins and software, as well as an implementation tool such as the consensus pathway (10), were recommended resources to help support audit execution. Technology that could assist in the reporting and collation of data was seen as a worthwhile investment to track changes in waste overtime. Participants were enthusiastic toward having the capability to input food waste data into an electronic system that would allow them to immediately, or retrospectively, access information to support decision making regarding patient care or foodservice improvements. Some foodservices were already achieving this through the use of an online portable patient intake application (visually estimating food waste on the ward when plates are collected by kitchen staff) (36) or sharing data with other foodservices and food rescue organisations. An electronic version of the consensus pathway food waste audit tool was recommended which would allow portability, accuracy, reduce paper waste and decrease time needed for manual data entry and analysis.

*“to my knowledge I haven’t found an easier way, and that’s where you’ve got systems like an electronic foodservice system such as mobile intake where you can just plug stuff in and then it gets extrapolated really easily, that obviously makes it a lot easier… so probably another enabler is if you’ve got an electronic food system, is there some way that that electronic food system can support the data entry for food waste? then that’s obviously an enabler that you can just pull data from.”* (Participant 17, Foodservice dietitian)

Many participants mentioned the conceivable financial incentives from reducing food waste as a result of actioning improvements to foodservice operations following audit data analysis. For example, potential cost savings could be generated through less waste hauling and reduction in resources such as time and labour for food preparation if there is a stronger understanding of portion requirements for service.

### 2. Change

Participants described that foodservice staff themselves were a barrier to food waste audit completion. Participants perceived that the magnitude of the change, and the time it required, would disrupt the daily routines of staff who are already resistant to the introduction of new initiatives. Some participants in manager roles alluded to the fact that some foodservice staff had been working in the same role and completing the same tasks for up to 40 years, emphasising why practice change may be difficult in this staff group. Managers also reported that foodservice staff perceived the extra work required for food waste audits being too difficult or not their responsibility. It was further reported that lower level of skills in data collection and limited knowledge of the importance of audits were barriers to accurate audit completion. Foodservice staff, although highly valued, were believed to be habitual in their tasks and impacted by any small or large variation in practice, which led to foodservice managers not wanting to involve them in planned food waste audits.

*“I just think that anything that disrupts the routine in the kitchen is burdensome for the staff and is seen as a burden for the staff. I don’t know what it is within the culture of the staff, but I think they don’t like anything that’s out of the ordinary to happen. They like to know their trolleys are going to be ready to go to the ward at a certain time, they take that up, when they’ve taken that up, they’ve got their break, then they come back. I just think in some ways, that becomes much more burdensome…”* (Participant 5, Foodservice dietitian)

Communicating to staff what the method is to complete food waste audits as well as ensuring the method was performed correctly were described as enablers. Many participants explained that improving the accuracy of audits could be achieved through planning, education and training. Participants provided unique examples of how staff are currently trained, and described they would use these strategies again. These included completing a trial audit, practicing audit methods, training manuals, paid training, and using materials provided by an in-house training advisor.

The most reiterated point in regards to education was to confirm that staff understand how to complete the task properly, so data are trustworthy. Checking with staff to confirm their awareness of the audit steps in the lead up to and during an audit as well as receiving their feedback on the process afterward was suggested by a number of participants. Conducting meetings with all foodservice staff completing the audit, and discussing the audit regularly, in as much detail as possible, was another strategy to ensure that *“everyone is on the same page.”* One participant (Participant 13, Sustainable food systems dietitian) highlighted that when communicating to specific staff groups with different levels of education, framing information with the appropriate lens was essential to ensuring the message was understood.

*“I think it all just generally falls back to training. And it needs to be robust training so that everyone knows exactly what needs to be done.”* (Participant 1, Support services manager)

Additionally, gaining foodservice staff buy-in to the idea of an audit before introducing it to their workload was viewed as a critical enabler. To reinforce staff interest one participant (Participant 17, Foodservice dietitian) suggested explaining to the staff group how the audit may benefit them in the future through possible additional funds being generated from less food waste, and that these funds could lead to the purchase of equipment which would make their job easier such as a new dishwasher. Gamification of the data collection process was another strategy said to possibly encourage staff participation whereby setting an achievable target or challenge for staff to work toward may make things fun. Participants also shared that they have staff members in their teams who are concerned about food waste and who would be intrinsically motivated to partake in an audit.

*“So I think definitely start at grassroots and talk to the staff about what they would think would be a benefit of a plate waste audit, or food waste audit, get their thoughts and ideas back, get an understanding of how they think they would like it to operate, and then obviously work with the managers on the other side of things.”* (Participant 2, Foodservice project officer)

Moreover, asking for staff to contribute to the audit design was a major recommendation from participants who believed that, *“foodservice staff have so much knowledge of the kitchen and how it works.”* (Participant 13, Sustainable food systems dietitian)

### 3. Processes, governance, and leadership

The hospital, as an organisation, was also perceived as a barrier toward conducting food waste audits. Participants described the initiation of food waste audits requiring planning and organisational support, however, staff hierarchy and task prioritisation reduced the endorsement of audits as a quality improvement project when compared to other clinically important tasks. Current waste management practices and the absence of guidelines or standards that mandated hospitals to measure food waste were identified as barriers to audit completion.

*“So to put on potentially other tasks, such as doing plate waste audits regularly, or fairly regularly, I think would be something difficult to get on board when we’re not even meeting accreditation standards, like choices with meals and things like that, which I think people would see and consider more of a priority than looking at food waste.”* (Participant 4, Food safety supervisor)

Several participants explained how different internal and external influences would help support the implementation of a food waste audit. One strategy described was the use of a top down approach from government to mandate food waste audits through policy or legislation and mandatory reporting. Similarly, participants mentioned that hospital level guidelines or standards are essential to follow for other tasks in the foodservice, such as nutrition standards for meals and menus, and having these for food waste audits would increase their regularity and support workflow.

*“And so if they make it in a way that every hospital mandates it, which I say if you can put it in a standard it will be perfect, because it makes it very clear that this has to be done.”* (Participant 10, Foodservice dietitian)

Executive level support from the organisation was said to be an enabler to implementing food waste audits. Some hospitals already had established environmental sustainability working groups and food waste audits were a “project” participants believed could be of future focus. Influencing change through identifying an individual in a leadership role (such as a supervisor, manager, or champion) was also discussed. Modifying other foodservice practices such as the foodservice type from cook chill to room service and when or how waste was collected were expected to facilitate the implementation of food waste audits and help achieve the overall goal of reducing food waste.

*“But there are other ways around meeting that waste management I guess. With a new food project that we’re going to introduce it will be designed so we can do plate waste audits, which is beneficial for a number of reasons. One of those reasons is obviously food waste data. The other reason is malnutrition of our patients and residents. So that gives dietitians sort of an insight or data to look into as far as how nutrition balances go for our patients. From our point of view, it gives us an insight of food waste.”* (Participant 11, Group management support services)

## Discussion

This study sought to identify the perspectives of staff involved in the operation of hospital foodservices on how an evidenced based consensus pathway food waste audit tool is perceived to translate into practice, and to determine the factors that influence the completion of food and food-related waste audits within this setting. With positive reflections toward the consensus tool presented by a majority of participants, hospital foodservices are encouraged to use the tool to plan, conduct and analyse a food and food related waste audit. Additionally, the findings indicate a number of reported barriers that are perceived to deter from prioritising and completing a food waste audit, including time, labour, resources, the staff population, hospital logistics, and change. However, various enabling factors were described by participants which present a solution to these barriers, such as outsourcing labour, training staff, organisational support, increased resources, and an uncomplicated audit procedure. For hospital foodservices to successfully trial or implement this tool in their practice the perspectives presented in this study should be reviewed. Appropriately applying the consensus pathway food waste audit tool may then promote possible outcomes such as decreases in waste, monetary savings and workflow enhancements. Moreover, if the use of the consensus tool is magnified to other food providing institutions experiencing high amounts of food waste such as aged care, prisons and childcare (37), this could have large influences on the economic, social and environmental impacts associated with food waste (38). This may promote reputational sustainability within these organisations or industries and (39) will contribute to the actions required to meet UN SDG 12.3 (3).

The consensus pathway food waste audit tool (10) may act as a guideline and reference point for hospital foodservices to recognise the essential decisions required before completing a food waste audit. The tool provides users with different choices to design an audit to best suit their foodservice operations. It appeared that the tool had face validity and was accepted by those working in the foodservice setting who are the intended users. Participants described the need to customise the tool to their needs and their contexts, and this is appropriate and recommended. A systematic review has found that providing flexibility in intervention design to different hospital sites that are incorporating the same intervention caters for unique site specific barriers and enablers (40). Additionally, transferring the current manual version of the tool to an online format which is interactive, similar to other foodservice innovations that can calculate food waste (36, 41–43), may be the next step to accelerate the tool’s accessibility and support its usage at scale. Refinements recommended in this study for the tool’s detail, design, and content could be integrated at that time. Furthermore, evidence based strategies to reduce food waste (8, 9) or waste management strategies aligned with the food recovery hierarchy (44) to divert waste from landfill could be built into the tool to provide direction on what actions can be taken after a waste audit is completed (45).

Within the three categories of (1) capacity, (2) change, and (3) processes, governance and leadership identified in this study the reported barriers and enablers were inter-linked, whereby collectively across all interviews the barriers to practice change were combatted by suggested enablers from the same or different participants (Figure 2). This pattern of clear associations between reported barriers and enablers occurred in a similar systematic review (40) exploring the barriers and enablers to implementing hospital interventions, which also found comparable themes to those derived from our data; system, staff, and the intervention itself. Participants in the present study suggested that for foodservices to support a food and food-related waste audit at their site they need to: increase overall staff resources; obtain necessary equipment; gain key stakeholder buy-in and executive support; develop an audit plan; and lead, communicate, and educate those expected to complete the audit. These could possibly be achieved through the involvement of hospital nutrition and dietetics departments in the audit process. Dietitians are equipped with communication, leadership and management skills, have experience with project development, implementation and evaluation (46), regularly have work-based placement students who could assist with data collection, and also collaborate with other professionals such as speech pathology (47) who have a vested interest in the foodservice. Furthermore there is a role for other staff groups, including members from the organisational environmental/sustainability committees, and nursing and medical staff who are committed to sustainability in healthcare (48).

### Limitations

This study was completed during the height of the delta-strain COVID-19 outbreak in Melbourne, Victoria (49). Consequently, recruitment of participants was challenging as the healthcare system prioritised its resources to combat the pandemic. There were considerably more participants in management roles rather than general foodservice roles, potentially due to their greater availability to participate in an online interview. However, although these staff were not front line workers they were still able to contribute valuable opinions regarding waste audit implementation within their teams. Completing interviews *via* Zoom (Version 5.5, Zoom video communications, California) rather than face to face may be viewed as a limitation, however, research has demonstrated interview participants perceive this method as time and cost-effective, convenient and practical (50). Furthermore, this method allowed the researchers to access previously out of reach participants as a result of the COVID-19 pandemic. This research has been designed in a way to support internal coherence, increasing its quality, trustworthiness and rigour (19). Philosophical alignment was established through the interpretivist paradigm whereby the authors’ relativist ontology, subjectivist epistemology, value bound axiology, use of qualitative description methodology and semi-structured interview data collection method supported research design (19, 51, 52). Additionally, reflexivity was demonstrated during data collection and analysis through explaining to participants the researcher’s connection to the project, and the research team conducting fortnightly peer-debriefing sessions where collective group discussion of themes occurred (29).

## Conclusion

This research uncovered perceptions of the factors that may influence implementation of food and food-related waste audits and an evidenced based consensus pathway food waste audit tool which could facilitate decision making among staff involved in hospital foodservices. The consensus tool appears to have face validity according to the participants interviewed in this study and could be used to design a food and food-related waste audit in a hospital foodservice. However, before integrating this tool into practice, hospital foodservices must consider the findings highlighted in this study to identify possible barriers and or enablers that may impact their site-specific style of food and food-related waste audit. Customisation of an audit best suited to a hospital’s environment, resources, workforce and perhaps behaviours will then support appropriate audit execution and outcomes.

## Data availability statement

Raw data is not available for sharing as ethics approval and participant consent does not allow for this.

## Ethics statement

The studies involving human participants were reviewed and approved by the Monash University Research Ethics Committee. The patients/participants provided their written informed consent to participate in this study.

## Author contributions

NC conducted the interviews, collated, analysed and interpreted the data, and wrote the manuscript. DG, JC, and JP supervised this process and critically reviewed the manuscript. All authors contributed to the conceptualisation and design of the study and have read and approved the final publication.

## Footnotes

1
https://otter.ai/
